# Thermal Viscoelasticity and Edge Integrity of Hydrophobic Acrylic Polymers Under Injector Compression

**DOI:** 10.3390/polym18202482

**Published:** 2026-10-11

**Authors:** Antonio de Nigris, Domenico Gentile, Tommaso Rossi, Luigi Ambrosone

**Affiliations:** 1 Department of Medicine and Health Sciences “V. Tiberio”, University of Molise, Via de Sanctis, 86100 Campobasso, Italy; a.denigris1@studenti.unimol.it (A.d.N.); domenico.gentile@unimol.com (D.G.); 2 IRCCS Fondazione G.B. Bietti, Via Santo Stefano Rotondo, 6, 00184 Rome, Italy; tommaso.rossi@fondazionebietti.it

**Keywords:** dynamic mechanical analysis, stress–dissipation relationship, hydrophobic acrylic intraocular lenses, edge fracture resistance, 46.35.+z, 46.70.-p, 62.20.M-, 83.80.Hj, 87.85.G-

## Abstract

The mechanical response of foldable intraocular lenses during injector-based delivery is governed by the relaxation dynamics of their polymer networks. To quantify this behavior, we performed a comprehensive mechanical characterization over the physiological temperature range and used the resulting relaxation parameters to construct a time–temperature equivalence model. By transforming the Williams–Landel–Ferry relation to use the viscoelastic transition temperature as reference and inverting it to match the strain rate imposed by the injector, we defined an equivalent temperature that represents the thermomechanical state experienced by the material during rapid folding. This parameter converts the deformation rate into an intuitive thermal quantity, revealing whether the polymer behaves in a rubbery, viscoelastic, or frozen regime. The equivalent temperature was evaluated across a wide range of optical powers and compared with the viscoelastic transition temperature to determine the thermomechanical safety margin of each material. The distance between these two temperatures identifies whether the polymer operates in a dissipative regime, where relaxation processes are fast enough to accommodate deformation, or in a transition-limited regime, where elastic energy accumulation and microfracture become more likely. The three investigated lens families exhibit distinct thermomechanical stratification: one group consistently remains well below its transition temperature, another shows a narrow but stable margin, and the third approaches its transition temperature during folding, indicating reduced relaxation capability and increased susceptibility to edge damage. Overall, the equivalent temperature framework provides a physically grounded and clinically useful tool for modeling the prediction of risk probability linked to mechanical damage during lens implantation. It also offers a practical guideline: pre-warming the lens to its equivalent temperature places the material in the same rheological condition that it would have under slow deformation, thereby reducing the likelihood of chipping before insertion.

## 1. Introduction

Hydrophobic acrylic intraocular lenses (IOLs) represent a distinctive class of lightly crosslinked polymer networks engineered to undergo large, reversible deformations during injector-based delivery [1]. Their mechanical response is governed by temperature-dependent viscoelastic relaxation, segmental mobility, and strain-rate sensitivity, making them an instructive model system for studying thermomechanical transitions in soft polymeric biomaterials. Although cataract surgery is performed more than 35 million times annually worldwide [2,3], the polymer physics underlying IOL folding remains insufficiently characterized from a materials-science perspective.

Modern IOLs are delivered through increasingly small corneal incisions (2.2–2.4 mm), requiring the optic to be folded and squeezed through a converging polymeric cartridge [4]. During this process, the lens experiences high curvature, localized bending, and rapid deformation, particularly along the lateral edge of the optic where stress concentration is maximal [5]. Several studies have reported the appearance of edge chipping, microcracks, or permanent indentations in specific hydrophobic acrylic formulations [6,7]. These observations suggest that the polymer network may temporarily enter a mechanically vulnerable state during folding, where its ability to dissipate strain energy is reduced.

Patient-specific anatomy, different histology of eye-bulb districts and severity of pathology to be treated state the physical requirements of ophthalmic artificial implants. Therefore, tuning the thermo-mechanical properties of biocompatible polymeric blends, such as acrylic-based devices, is crucial in determining implant success and anatomical stability [8,9,10]. Hydrophobic acrylics exhibit a broad glass-transition interval and a pronounced temperature dependence of their viscoelastic moduli [11,12,13]. Because IOLs are typically stored at low temperature and handled at approximately 20 °C in the operating room, their mechanical state during folding corresponds to the early rubbery regime, where segmental mobility is only partially activated. Dynamic Mechanical Analysis (DMA) provides access to the storage modulus $E^{\prime }$ and loss modulus $E^{\prime \prime }$, enabling a quantitative assessment of the elastic and dissipative components of the response [12]. The viscoelastic transition temperature $T_{c}$, defined as the point where $E^{\prime }\approx E^{\prime \prime }$, marks the crossover between viscous-dominated and elastic-dominated behavior [13]. Materials with $T_{c}$ above the injection temperature remain relatively stiff and accumulate elastic energy during bending, whereas materials with lower $T_{c}$ retain higher dissipation and reduced stress concentration.

However, temperature alone does not fully determine the mechanical state of the polymer during folding. Injector-based delivery imposes a rapid deformation that can effectively shift the polymer toward a stiffer thermomechanical state. This strain-rate sensitivity can be described using the Williams–Landel–Ferry (WLF) equation, which relates temperature to relaxation time in amorphous polymers. By inverting the WLF relation and using $T_{c}$ as reference, we define an equivalent temperature $T_{eq}$ that quantifies the thermomechanical state induced by the imposed strain rate. In this framework, rapid folding corresponds to a positive thermal shift, effectively “cooling” or “warming” the polymer depending on its relaxation dynamics.

Beyond its relevance for ophthalmic applications, the present work introduces a general thermomechanical framework for lightly crosslinked polymers subjected to rapid strain-rate-controlled deformation. By combining DMA-derived relaxation parameters, WLF time–temperature superposition, and an analytical strain-rate model, the approach provides a quantitative route for predicting failure-prone conditions in soft polymer networks. This methodology is not restricted to IOL materials and can be applied to any polymeric system where deformation rate and thermal history jointly determine the mechanical state.

Building on these concepts, the present study combines DMA measurements with analytical modeling to establish a quantitative link between viscoelastic relaxation, strain-rate effects, and the propensity for edge chipping in hydrophobic acrylic IOLs. First, we characterize three commercial material families over the 25–40 °C range to determine their dynamic moduli, dissipation behavior, and viscoelastic transition temperature. Second, we develop a strain-rate-based mechanical model that incorporates injector geometry, dioptric-dependent thickness, and WLF-derived relaxation times. By integrating these approaches, we introduce a dimensionless chipping index, a measure of predicted susceptibility, that captures the balance between imposed deformation rate and intrinsic relaxation capability. This unified framework provides a mechanistic interpretation of edge damage in hydrophobic acrylic IOLs and offers design criteria for safer polymer formulations and delivery systems.

The interplay between $T_{eq}$ and $T_{c}$ provides a physically meaningful measure of the material’s strain-rate sensitivity. While $T_{c}$ identifies the intrinsic viscoelastic transition of the polymer network, $T_{eq}$ describes the effective thermomechanical state reached during rapid folding. The distance between these two temperatures determines whether the polymer operates in a dissipative regime, where relaxation processes are fast enough to accommodate deformation, or in a transition-limited regime, where elastic energy accumulation becomes significant. This conceptual framework establishes the basis for interpreting the thermomechanical behavior of different IOL materials and for identifying conditions under which edge damage is more likely to occur.

## 2. Theoretical Aspects

The probability of edge chipping in an intraocular lens (IOL) depends on both the intrinsic nature of the polymeric material and the mechanical solicitation applied during injection. The material acts as the predisposing factor, whereas the imposed mechanical load represents the triggering factor. The polymer establishes the maximum amount of energy that the lens edge can absorb before failure, while the way the IOL is inserted into the cartridge and folded determines the geometric stress concentration at the edge. Based on these considerations, we define the chipping index as

(1) $$I_{s}=\frac{{\dot{\varepsilon }}_{\mathrm{imposed}}}{{\dot{\varepsilon }}_{\mathrm{limit}}}$$

where ${\dot{\varepsilon }}_{\mathrm{imposed}}$ is the strain rate imposed by the surgeon through the injector, and ${\dot{\varepsilon }}_{\mathrm{limit}}$ is the maximum strain rate that the material can dissipate without accumulating elastic energy. The chipping index $I_{s}$ quantifies the ratio between the externally imposed deformation rate and the intrinsic viscoelastic relaxation capability of the material. When $I_{s}>1$, the imposed deformation exceeds the dissipative capacity of the polymer, generating internal stresses that may lead to edge chipping. The imposed strain rate is not simply the ratio “displacement/time”, but depends on the conical geometry of the injector [14]:

(2) $${\dot{\varepsilon }}_{\mathrm{imposed}}=\frac{\varepsilon_{\mathrm{geom}}}{\Delta t_{\mathrm{inj}}}$$

where $\varepsilon_{\mathrm{geom}}$ is the geometric deformation induced by the injector and $\Delta t_{\mathrm{inj}}$ is the transient injection time calculated from realistic scenarios [15,16]. The injector tip is a converging conical duct with inlet diameter $d_{\mathrm{in}}$ and outlet diameter $d_{\mathrm{out}}$. During the forward motion imposed by the plunger, the IOL undergoes a three-dimensional squeezing. The nominal radial compression strain is

(3) $$\varepsilon_{\mathrm{rad}}=\ln\;\left(\frac{d_{\mathrm{in}}}{d_{\mathrm{out}}}\right)$$

The real system, however, exhibits thickness and curvature that depend on the dioptric power *D* of the lens. The material shape factor is defined as

(4) $$\Phi \left(D\right)=\frac{h_{\mathrm{center}}\left(D\right)}{h_{\mathrm{edge}}\left(D\right)}$$

where $h_{\mathrm{center}}$ and $h_{\mathrm{edge}}$ denote the central and edge thicknesses, respectively. To determine the central thickness, the refractive index *n* must be considered:

(5) $$h_{\mathrm{center}}=h_{\mathrm{edge}}+\frac{D{\left(\frac{d}{2}\right)}^{2}}{2\left(n-n_{\mathrm{med}}\right)}$$

where *D* is the dioptric power, *d* the optical diameter, *n* the refractive index of the material, and $n_{\mathrm{med}}$ the refractive index of the surrounding aqueous medium. This formulation is based on a paraxial spherical approximation; real aspheric designs are not considered in this work. Thus, the geometric strain tensor, scaled by the shape factor, becomes

(6) $$\varepsilon_{\mathrm{geom}}=\Phi \left(D\right)\;\varepsilon_{\mathrm{rad}}$$

To determine ${\dot{\varepsilon }}_{\mathrm{limit}}$, the rheological properties of the polymer must be considered. We employ the Williams–Landel–Ferry (WLF) theory [17], which states that the thermal shift factor $a_{T}$, defined as the ratio between the molecular relaxation times at temperature *T* and at a reference temperature $T_{\mathrm{ref}}$,

(7) $$a_{T}=\frac{\tau (T)}{\tau (T_{\mathrm{ref}})}$$

is given by

(8) $$\log_{10}a_{T}=\frac{-C_{1}\;\left(T-T_{r}\right)}{C_{2}+\left(T-T_{r}\right)}$$

where $C_{1}$ and $C_{2}$ are empirical constants depending on the polymer. Within the validity of WLF theory, the maximum strain rate that the material can tolerate before losing viscous dominance is

(9) $${\dot{\varepsilon }}_{\mathrm{limit}}=\omega_{0}\;a_{T}$$

where $\omega_{0}$ is the reference angular frequency (at fixed frequency of 1 Hz in our DMA measurements). Equations (1)–(9) allow the computation of the chipping index $I_{s}$ and the identification of three distinct mechanical regimes during injection:

*Safe regime*: $I_{s}<0.75$. The imposed strain rate is well below the material limit, and the polymer remains in a viscous-dominant state. Edge chipping is unlikely.
*Transition regime*: $0.75\leq I_{s}\leq 1.25$. The material approaches its viscoelastic transition. Small perturbations (temperature fluctuations, manual bumps) may trigger micro-damage. Risk is moderate.
*Chipping regime*: $I_{s}>1.25$. The imposed strain rate exceeds the relaxation capability of the polymer. The material behaves elastically and cannot dissipate energy efficiently, leading to highly predicted susceptibility toward edge chipping.

This division of the $\left(T,I_{s}\right)$ plane into three regions of mechanical severity is based on viscoelastic data and experimental bending results. The region $I_{s}<0.25$ corresponds to conditions in which the material is fully relaxed and no damage is observed. The interval $0.75\leq I_{s}\leq 1.25$ represents the transition zone, where the response depends strongly on the proximity to $T_{c}$ and on the lens geometry. Finally, $I_{s}\geq 1.25$ identifies conditions in which the mechanical severity exceeds the material’s dissipative capacity, leading to the systematic appearance of chipping and microcracks.

This division of the $\left(T,I_{s}\right)$ plane into three operational regions of mechanical severity is rooted in polymer viscoelasticity theory and experimental injector dynamics [5,6]. The threshold $I_{s}=1.0$ represents the theoretical boundary where the imposed strain rate matches the maximum relaxation capacity of the polymer network (${\dot{\varepsilon }}_{\mathrm{imposed}}={\dot{\varepsilon }}_{\mathrm{limit}}$) [17]. The interval $0.75\leq I_{s}\leq 1.25$ defines a ±25% transition band that accounts for manual surgical injection rate fluctuations, cartridge alignment variations, and localized stress concentration at the optic edge. Specifically:

*Safe regime* ($I_{s}<0.75$): The imposed strain rate is comfortably below the dissipation limit of the polymer. Chain relaxation is fast enough to accommodate deformation without accumulating residual stress, rendering edge damage unlikely.
*Transition regime* ($0.75\leq I_{s}\leq 1.25$): The material approaches its viscoelastic crossover ($E^{\prime }\approx E^{\prime \prime }$). Small perturbations (such as thermal drops or plunger speed spikes) can trigger localized strain energy buildup, representing a moderate failure risk zone.
*Chipping regime* ($I_{s}>1.25$): The imposed deformation rate substantially exceeds the viscoelastic relaxation spectrum of the material. The polymer network behaves elastically, accumulating unrelaxed stress that promotes microcracking and edge chipping during cartridge passage [5].

## 3. Experimental

### 3.1. Materials

Three commercially available hydrophobic acrylic intraocular lenses (IOLs) were selected to represent distinct polymer formulations and manufacturing approaches routinely used in cataract surgery. Physiol Micropure 123^®^ (BVI Medical, Rome, Italy) (MCP) lenses are composed of a hydrophobic acrylic copolymer based on HEMA/PEA/PPGDMA and feature a symmetric biconvex monofocal geometry. TECNIS^®^ 1-piece (Johnson & Johnson Vision Care, Pomezia, Rome, Italy) (TEC) lenses consist of a hydrophobic acrylic copolymer with a biconvex profile and are supplied fully hydrated. Both MCP and TEC lenses were stored at room temperature in physiological saline solution (NaCl, 0.9%) until testing; immediately before measurement, excess surface liquid was gently removed using lint-free filter paper to ensure reproducible contact conditions. AcrySof Clareon^®^ (Alcon, Rome, Italy) (ACS) lenses, made of a hydrophobic acrylate–methacrylate copolymer and characterized by an asymmetric biconvex anterior geometry, are provided dry-packed. These lenses were stored at room temperature in their original packaging and tested without hydration to preserve their native mechanical state. All samples were handled in such a way as to avoid unintended mechanical preloading or surface damage.

### 3.2. Methods

**Figure 1:**
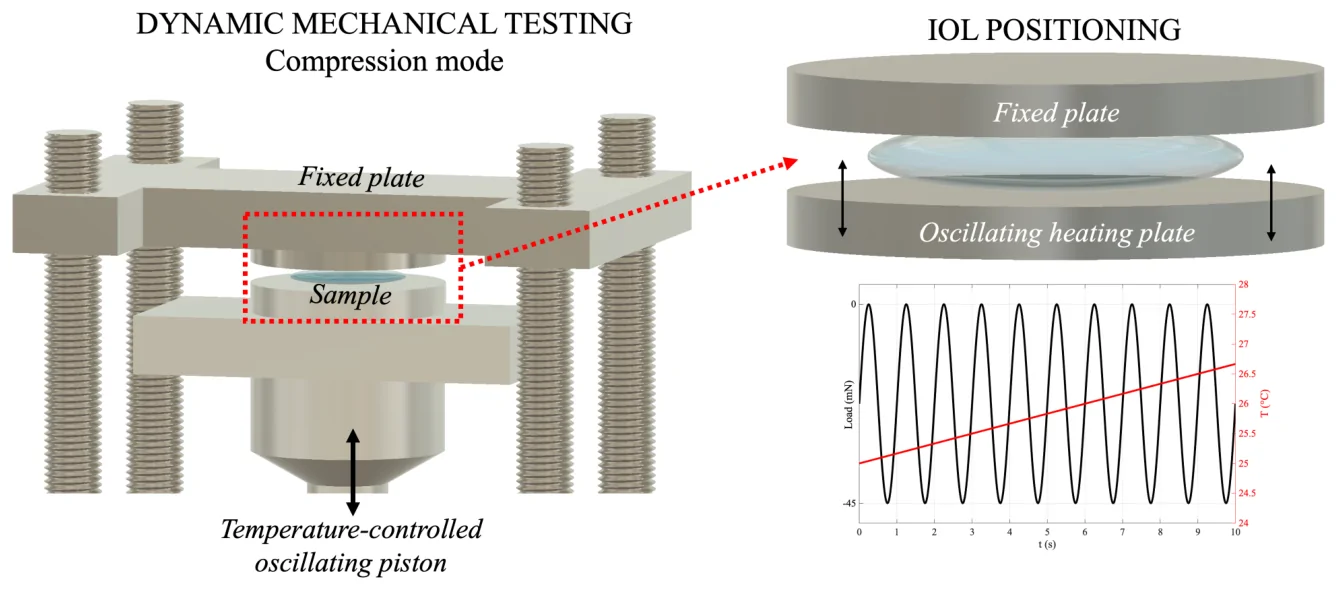
Schematic representation of the experimental setup used for dynamic mechanical analysis (DMA) of the intraocular lenses. The system includes the temperature-controlled chamber (25–40 °C), the oscillatory loading module operating at 1 Hz and 45 mN, and the displacement sensor used to extract the viscoelastic response.

Dynamic mechanical analysis was performed using a DMA 1 analyzer (Mettler Toledo, Greifensee, Switzerland). The instrument operates over a broad temperature range (25 °C to 600 °C) with a thermal accuracy of ±0.75 K and allows the application of dynamic loads between 0.001 N and 10 N, frequencies from 0.001 to 300 Hz, and displacements up to ±1.0 mm. Data acquisition and processing were carried out using the STAR^e^ software (V21.0) package. For thermal–mechanical characterization, each IOL was mounted inside the insulated chamber using the compression configuration with cylindrical plates (10 mm diameter). This configuration ensures uniform contact and minimizes edge effects, enabling accurate extraction of the viscoelastic moduli. All measurements were performed in dynamic load-control mode by applying a sinusoidal compressive load of 45 mN (corresponding to a pressure of approximately 12 mmHg) at a fixed frequency of 1 Hz. These conditions were selected to mimic the low-frequency mechanical environment relevant to injector-based folding while ensuring high signal-to-noise ratio in the viscoelastic response. The chamber temperature was increased from 25 °C to 40 °C following a controlled heating ramp of 1 °C min^−1^. This range spans the transition from room-temperature handling to physiological conditions and allows identification of the viscoelastic transition temperature $T_{c}$ for each material. No pre-load was applied prior to testing to avoid introducing residual stresses or altering the native mechanical state of the lenses. During each measurement, the instrument recorded the applied force, resulting displacement, phase lag, and derived viscoelastic quantities including storage modulus $E^{\prime }$, loss modulus $E^{\prime \prime }$, complex modulus $E^{*}$, loss tangent tanδ, and the corresponding dissipative parameters. Due to the restricted availability and high costs of clinical-grade intraocular lens implants, experimental testing was performed on individual representative lenses across different dioptric power rather than large sample replicates. Measurement reliability and variability were addressed by considering the intrinsic accuracy ofthe DMA instrument (±0.75° for temperature and ±1% for dynamic moduli). Experimental uncertainties were propagated through using standard error propagation methods to evaluate the confidence limits of $T_{eq}$ and the chipping index $I_{s}$ [18]. The setup is reported in Figure 1. It is worth mentioning that DMA measurements provide useful insights about temperature-dependent viscoelasticity of complex polymeric matrices. However, the setup adopted still represents an approximation of multi-variable real-life scenarios which involve the IOL injection, whose kinetics depends on the operator. Nevertheless, although the DMA analysis with fixed frequency and load does not faithfully reproduce the complexity of the phenomenon, it is a great trade-off between high accuracy and complete understanding of the mechanical deformations because external solicitations in the oscillatory regime are capable to trigger the time- and temperature-resolved relaxation of the fibers.

## 4. Results and Discussion

**Figure 2:**
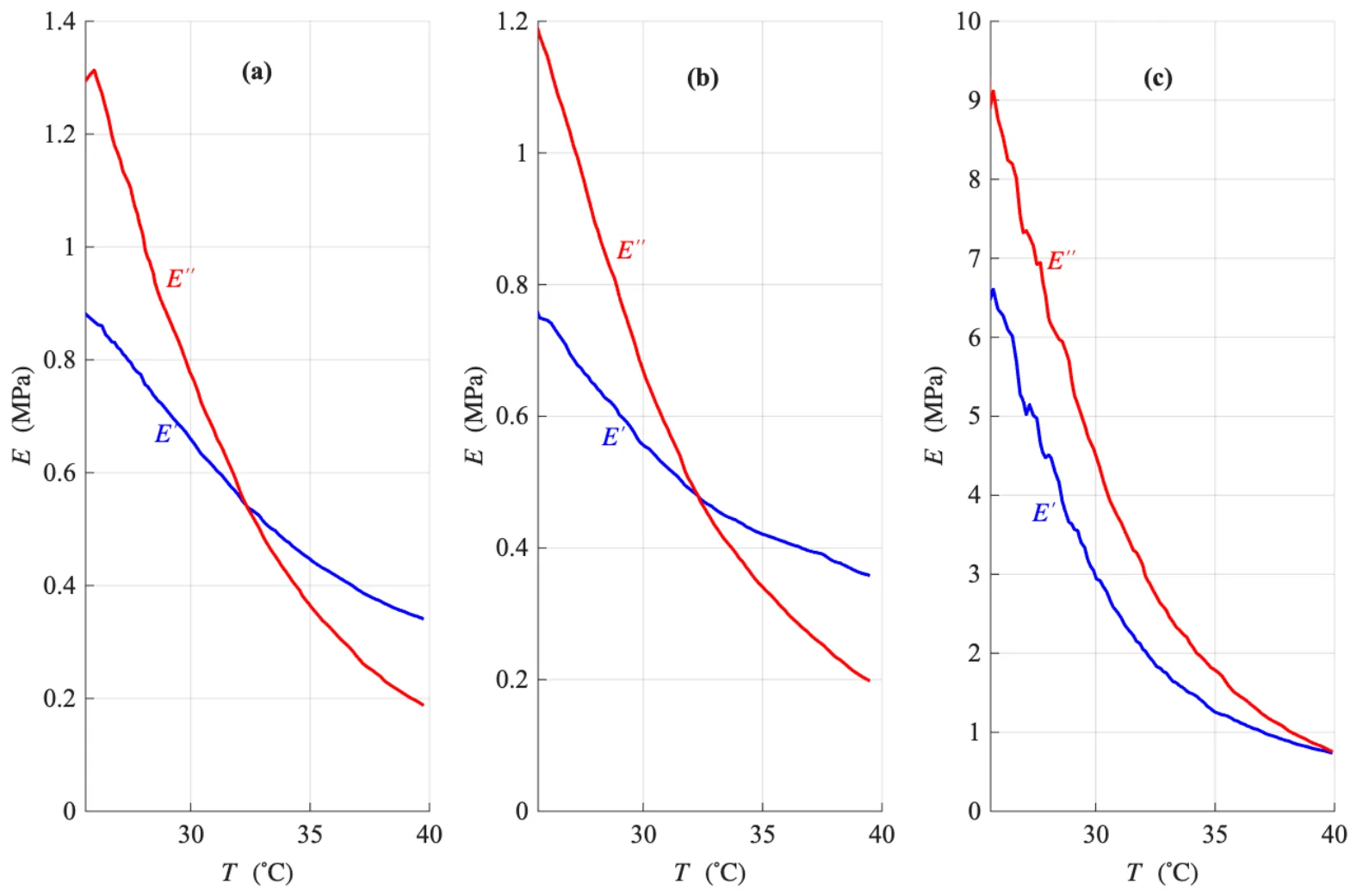
Temperature-dependent storage modulus $E^{\prime }\left(T\right)$ and loss modulus $E^{\prime \prime }\left(T\right)$ obtained by DMA for the three hydrophobic acrylic IOL families investigated. (**a**) ACS: viscoelastic moduli of the ACS material. (**b**) MCP: corresponding $E^{\prime }\left(T\right)$ and $E^{\prime \prime }\left(T\right)$ curves for the MCP material. (**c**) TEC: viscoelastic response of the TEC material. All measurements were performed between 25 and 40 °C under a sinusoidal load of 45 mN. Uncertainty in $T_{c}$ determination corresponds to the thermal resolution of the instrument (±0.75 °C).

Figure 2 shows the temperature-dependent storage modulus $E^{\prime }\left(T\right)$ and loss modulus $E^{\prime \prime }\left(T\right)$ measured by DMA in the range 25–40 °C for ACS (+21.5 D), MCP (+21 D), and TEC (+21 D). All hydrophobic acrylic IOL materials exhibit the same qualitative trend: both moduli decrease with temperature, but $E^{\prime \prime }\left(T\right)$ decays much more rapidly than $E^{\prime }\left(T\right)$. As a consequence, a critical temperature $T_{c}$ exists at which the two curves intersect ($E^{\prime }=E^{\prime \prime }$). This analysis was performed for all samples, and the resulting $T_{c}$ values are collected in Table 1. The presence of a well-defined crossover naturally partitions the (T,E) plane into two distinct mechanical regimes. Hydrophobic acrylics possess a very low glass transition temperature $T_{g}$, typically between 11 and 15 °C, consistent with the broad glass-transition interval characteristic of lightly crosslinked acrylic networks [19,20]. At 20 °C, representative of operating-room conditions, the material resides in the early region of its rubbery phase, still very close to the end of its glass-transition interval. In this state, the elastic modulus $E^{\prime }$ remains relatively high and the segmental mobility of the polymer chains is only partially developed. In the domain $T<T_{c}$, the viscoelastic response is dominated by the viscous component ($E^{\prime \prime }>E^{\prime }$). Polymer chains slide past one another with substantial internal friction, and most of the mechanical energy applied by the sinusoidal load is dissipated as heat rather than stored elastically. The material behaves as a highly viscous system with limited ability to accumulate elastic energy, in agreement with classical interpretations of the α-relaxation regime [21]. In the domain $T>T_{c}$, both moduli continue to decrease with temperature, but $E^{\prime \prime }$ drops much more steeply than $E^{\prime }$. The reduction in internal friction allows freer segmental motion; the ability to dissipate energy declines rapidly, while the elastic contribution, although reduced, remains sustained by the chemical crosslinking of the copolymer network. Above $T_{c}$, the material behaves as a rubbery elastic solid ($E^{\prime }>E^{\prime \prime }$), completing the α-relaxation process that begins at 11–15 °C. This relaxation is essentially exhausted between 37 and 40 °C, depending on the material family, consistent with the temperature range in which hydrophobic acrylics reach full rubbery stabilization [22]. From an application standpoint, this behavior is ideal. At room temperature the IOL is soft, highly deformable, and easily foldable for injector-based delivery. Once implanted and warmed to physiological temperature (above $T_{c}$ for ACS and MCP), the lens regains the elastic stability required to maintain its designed geometry. Measurements performed on moist IOL rather than dry-packed lenses are expected to present noticeable variations on the mechanical response after compression. However, TEC (wet) is the only kind of lens whose behaviour differs significantly from the other samples. That is to say, MCP (wet) and ACS (dry) present similar relaxation dynamics, suggesting that the cross-over point below 35 °C is intrinsic of the IOL acrylic nature and is not actively influenced by saline accumulation on the lens surface. Indeed, the DMA analysis might not be able to sense slight mechanical variations which involve the external layers of the moist hydrophobic IOLs. The magnitude of the compression penetrates a sufficiently deep portion of the lens volume and reduces the sensitivity towards possible edge effects.

**Figure 3:**
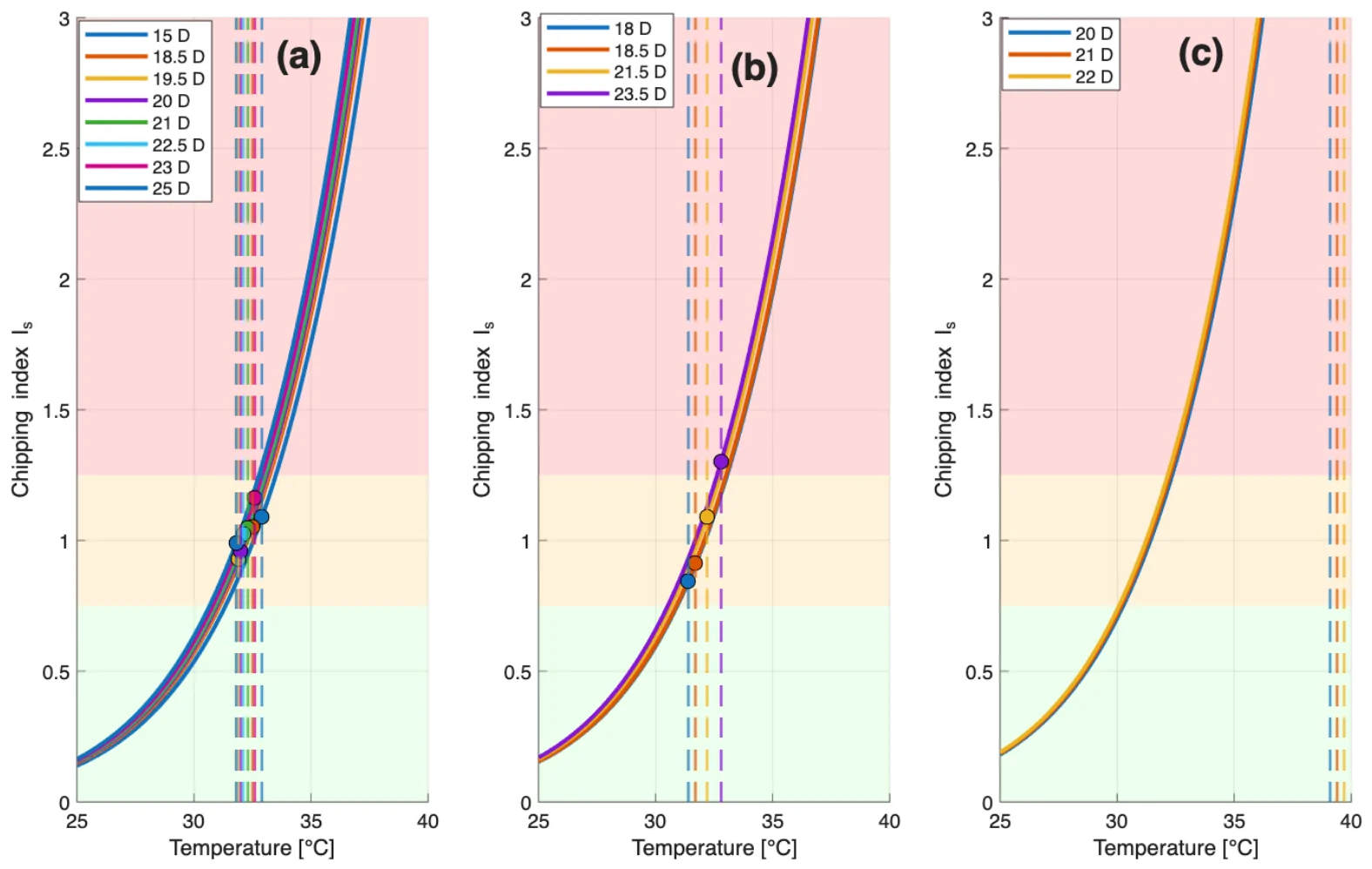
Temperature-dependent chipping index $I_{s}\left(T\right)$ for the three investigated IOL material families: (**a**) ACS, (**b**) MCP, and (**c**) TEC. Each panel reports the curves corresponding to the optical powers *D* listed in the legend, together with the viscoelastic transition temperature $T_{c}$ indicated by vertical dashed lines of matching color. The background shading highlights the operational chipping domains: safe (green), uncertain (yellow), and failure-dominated (red). ACS and MCP lenses predominantly lie near the safe–uncertain boundary, whereas TEC lenses enter the failure region due to their higher stiffness and reduced viscous dissipation at physiological temperature.

DMA measurements were performed under a sinusoidal load of 45 mN, corresponding to an intraocular pressure of approximately 12 mmHg, well within the physiological range of 10–21 mmHg [23]. The observed decrease in the viscoelastic moduli therefore reflects the intrinsic mechanical relaxation of the material as it equilibrates under a realistic ocular pressure while approaching body temperature. In other words, the DMA results capture how hydrophobic acrylic IOLs complete their α-relaxation and achieve mechanical stabilization precisely during the thermal transition experienced upon implantation. As extracted from Table 1, the transition temperature $T_{c}$ of the ACS and MCP lenses lies approximately 10–15 °C above the operating-room temperature (20 °C). Consequently, the empirical surgical practice of pre-warming the IOL effectively reduces or eliminates this thermal gap, shifting the polymer into the elastic regime where $E^{\prime \prime }<E^{\prime }$. In this state, the acrylic network bends without micro-cracking, ensuring safe folding and injection. This practical observation is fully consistent with the viscoelastic trends measured by DMA, which show that once the material is brought closer to or above $T_{c}$, viscous dissipation rapidly decreases and the elastic response of the crosslinked network becomes dominant. To translate the viscoelastic parameters of Table 1 into operational model-based chipping-risk maps, the temperature-dependent chipping index $I_{s}\left(T\right)$ was computed for all investigated IOL families. The analysis was carried out under oscillatory loading at 1 Hz, corresponding to an angular frequency $\omega_{0}=6.28\;\mathrm{rad}\;s^{-1}$. The optical thickness was set to d=6mm, with refractive indices $n_{\mathrm{med}}=1.336$ for the surrounding medium and material-specific values n=1.55 (ACS), n=1.52 (MCP), and n=1.47 (TEC). The injector was modeled as a conical channel with $d_{\mathrm{in}}=3.0\;\mathrm{mm}$ and $d_{\mathrm{out}}=2.3\;\mathrm{mm}$. Temperature dependence was introduced through the Williams–Landel–Ferry shift factor using the universal constants $C_{1}=9.5$ and $C_{2}$ = 65 °C at the operating-room reference temperature $T_{Rif}$ = 20 °C, consistent with the glass-transition behavior of commercial hydrophobic acrylics [24,25]. Figure 3 displays the resulting chipping domains for ACS, MCP, and TEC lenses. ACS and MCP materials predominantly lie at the boundary between the safe (green) and uncertain (yellow) regions, with $I_{s}\left(T\right)$ values clustered around the transition threshold. This behavior reflects their moderate elastic modulus at physiological temperature and their viscoelastic transition temperatures $T_{c}$ ≈ 32 °C, which place both families above the transition at 37 °C. In this partially relaxed regime, the polymer network dissipates a substantial fraction of the strain rate imposed by the injector. Furthermore, the higher refractive indices of ACS (n=1.55) and MCP (n=1.52) reduce the geometric stress amplification term ${\left(n-n_{\mathrm{med}}\right)}^{-1}$, limiting the bending stress at the optic edge. As a result, ACS and MCP lenses do not enter the failure-dominated domain under standard conditions, although they remain sensitive to variations in plunger speed, temperature, and optical power. In contrast, TEC lenses consistently reach the red failure region, indicating a markedly higher propensity for edge chipping. Three mechanisms contribute synergistically to this behavior. First, TEC lenses exhibit the lowest refractive index (n=1.47), which maximizes the geometric stress term and increases the bending moment at the lens edge. Second, their viscoelastic transition temperature ($T_{c}\approx 39$–40 °C) lies above physiological temperature, meaning that TEC lenses operate in a predominantly elastic, low-dissipation regime during injection.

Overall, Figure 3 reveals a clear mechanical separation among the three material families. ACS and MCP operate near the safe–uncertain boundary, whereas TEC lenses enter the failure-dominated domain, providing a quantitative explanation for their greater susceptibility to edge chipping during injector-mediated folding. The DMA results of Figure 2 show that, under the rapid axial compression imposed by the injector, the polymer chains attempt to dissipate energy through the loss modulus $E^{\prime \prime }$, but they cannot reorganize or relax in a coordinated manner. When the imposed strain rate is extremely high (${\dot{\varepsilon }}_{imposed}\gg 1\;s^{-1}$), the molecular network does not have sufficient physical time to flow or rearrange. As a consequence, the chains respond in a progressively stiffer manner, as if they were “frozen” on the timescale of deformation. This behavior is the fundamental physical basis of the time–temperature equivalence principle [17]. In order to quantify how the injector modifies the molecular relaxation times, we introduce the thermal shift factor $a_{T}$, which expresses how many decades the relaxation spectrum must be shifted to match the imposed strain rate. For example, if the injector operates at ${\dot{\varepsilon }}_{imposed}=62.8\;s^{-1}$ while the DMA test is performed at $\omega_{0}=6.28\;\mathrm{rad}/s$, a shift factor $a_{T}=10$ indicates that the polymer is forced to respond one decade faster than under laboratory conditions. In other words, the deformation is so rapid that the chains cannot physically relax, and the material behaves as if it were at a lower effective temperature. To map the injector kinematics onto the DMA data, it is convenient to adopt a WLF formulation using the viscoelastic transition temperature $T_{c}$ as the reference. When the reference temperature is shifted from $T_{\mathrm{rif}}$ to $T_{c}$, the WLF coefficients transform according to the rheological consistency relations [26,27], i.e.,

(10) $$C_{1}^{c}=\frac{C_{1}\;C_{2}}{C_{2}+\left(T_{c}-T_{\mathrm{rif}}\right)},\;C_{2}^{c}=C_{2}+\left(T_{c}-T_{\mathrm{rif}}\right),$$

where $T_{\mathrm{rif}}$ = 20 °C corresponds to the operating-room temperature. By inverting the WLF equation with $T_{c}$ as reference, the equivalent temperature $T_{eq}$ associated with the imposed strain rate is obtained as,

(11) $$T_{eq}=T_{c}-\frac{C_{2}^{c}\;\log_{10}\;\left(\frac{{\dot{\varepsilon }}_{imposed}}{\omega_{0}}\right)}{C_{1}^{c}+\log_{10}\;\left(\frac{{\dot{\varepsilon }}_{imposed}}{\omega_{0}}\right)}.$$

**Figure 4:**
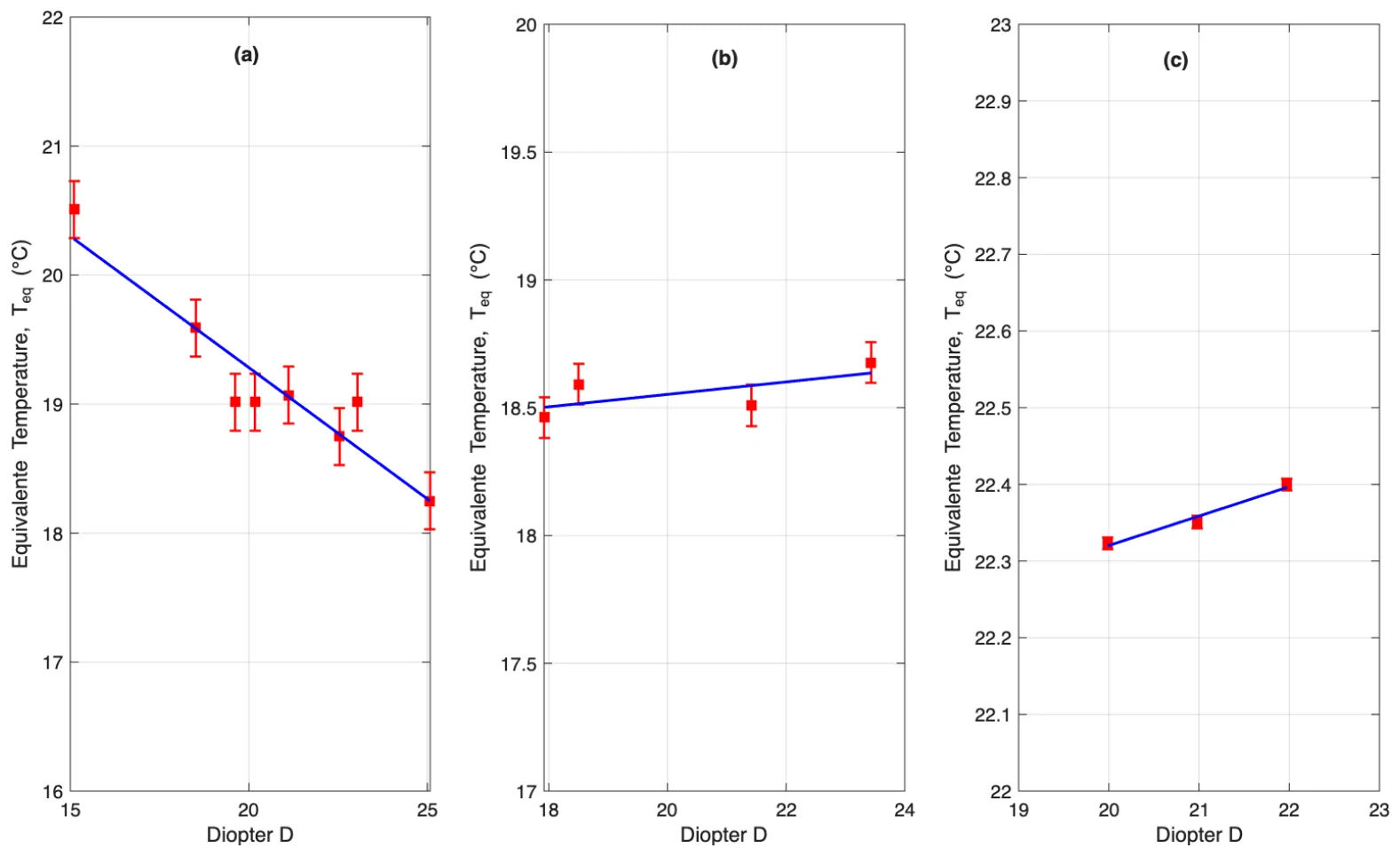
Equivalent temperature $T_{eq}$ as a function of dioptric power *D* for the three investigated IOL families: (**a**) ACS, (**b**) MCP, and (**c**) TEC. ACS lenses show a marked decrease of $T_{eq}$ with increasing *D* ($T_{eq}=-0.20D+23.28$), indicating stronger viscoelastic penalization at higher optical powers. MCP lenses exhibit an almost flat trend ($T_{eq}=0.02D+18.14$), revealing minimal sensitivity to geometry. TEC lenses display a mild positive slope ($T_{eq}=0.04D+21.54$), suggesting that higher powers shift the material toward warmer equivalent conditions, closer to the elastic regime.

The quantity $T_{eq}$ represents a virtual working temperature: it indicates the rheological state that the polymer would exhibit if the deformation were applied slowly, rather than under the rapid strain rate imposed by the injector. A high $T_{eq}$ (close to 30–35 °C) implies that the combination of dioptric power and strain rate is gentle enough for the material to behave in a fully rubbery/viscous manner, ensuring safe folding. Conversely, when $T_{eq}$ approaches the experimental $T_{c}$, the injector strain rate effectively “consumes” the thermal safety margin of the polymer, pushing it toward the viscoelastic transition where microfracture and chipping may occur. In practical terms, $T_{eq}$ converts a kinematic variable that is difficult to visualize (the imposed strain rate) into an immediate thermomechanical indicator of safety. If the surgeon pre-warms the IOL to $T_{eq}$, the material is brought into the same rheological condition that it would have under a slow deformation, thereby reducing the probability of risk of chipping during injection. Using Equation (10) and Equation (11), the $T_{eq}$ was computed for all IOLs and is reported in Table 1. The values of $T_{eq}$ are displayed in Table 1 and plotted in Figure 4, which summarizes the dependence of the equivalent temperature on dioptric power for each material family. The linear fits highlight a material-dependent trend, where the reported uncertainties correspond to the standard errors of the fitted coefficients:

$$\begin{array}{ccc} T_{eq}^{\mathrm{ACS}} & =(-0.20\pm 0.07)D+(23\pm 2),\; & \left(R^{2}=0.90\right)\\ T_{eq}^{\mathrm{MCP}} & =(0.02\pm 0.02)D+(18\pm 2),\; & \left(R^{2}=0.60\right)\\ T_{eq}^{\mathrm{TEC}} & =(0.04\pm 0.02)D+(22\pm 1),\; & \left(R^{2}=0.98\right) \end{array}$$

The near-zero slopes for MCP and TEC, combined with their low/high $R^{2}$ values, confirm that $T_{eq}$ is practically independent of dioptric power for these materials. Conversely, the significant negative slope for ACS reflects a clear diopter-dependent decrease in $T_{eq}$.

As shown in Figure 4, error bars representing the propagated instrumental uncertainty (derived from DMA measurement tolerances of ±0.75 °C in temperature and ±1% in dynamic force) have been incorporated into the plot. The confidence intervals of the linear fits confirm that the negative slope for ACS remains statistically distinct from the flat behavior of MCP and TEC, demonstrating the reliability of the dioptric power trends despite sample availability constraints inherent to clinical-grade implants.

For ACS lenses, the negative slope indicates that higher dioptric powers drive the system towards lower equivalent temperatures, i.e., further away from the viscoelastic transition region. This implies that strongly powered ACS lenses experience a more penalizing mechanical condition during injector-based folding and therefore benefit the most from careful pre-warming to ensure that the material operates in the elastic regime without chipping. In contrast, MCP lenses exhibit an almost vanishing slope, revealing that $T_{\mathrm{eq}}$ is essentially insensitive to optical power. This behavior suggests that the viscoelastic response of MCP lenses is dominated by the intrinsic polymer properties rather than by geometry, making their folding behavior highly predictable across the entire dioptric range. TEC lenses, on the other hand, show a slightly positive slope, meaning that higher dioptric powers shift $T_{\mathrm{eq}}$ towards higher values, closer to the elastic regime. As a consequence, low-power TEC lenses represent the most critical configuration in terms of probability of chipping risk, whereas high-power TEC lenses are comparatively less demanding from a viscoelastic standpoint.

**Figure 5:**
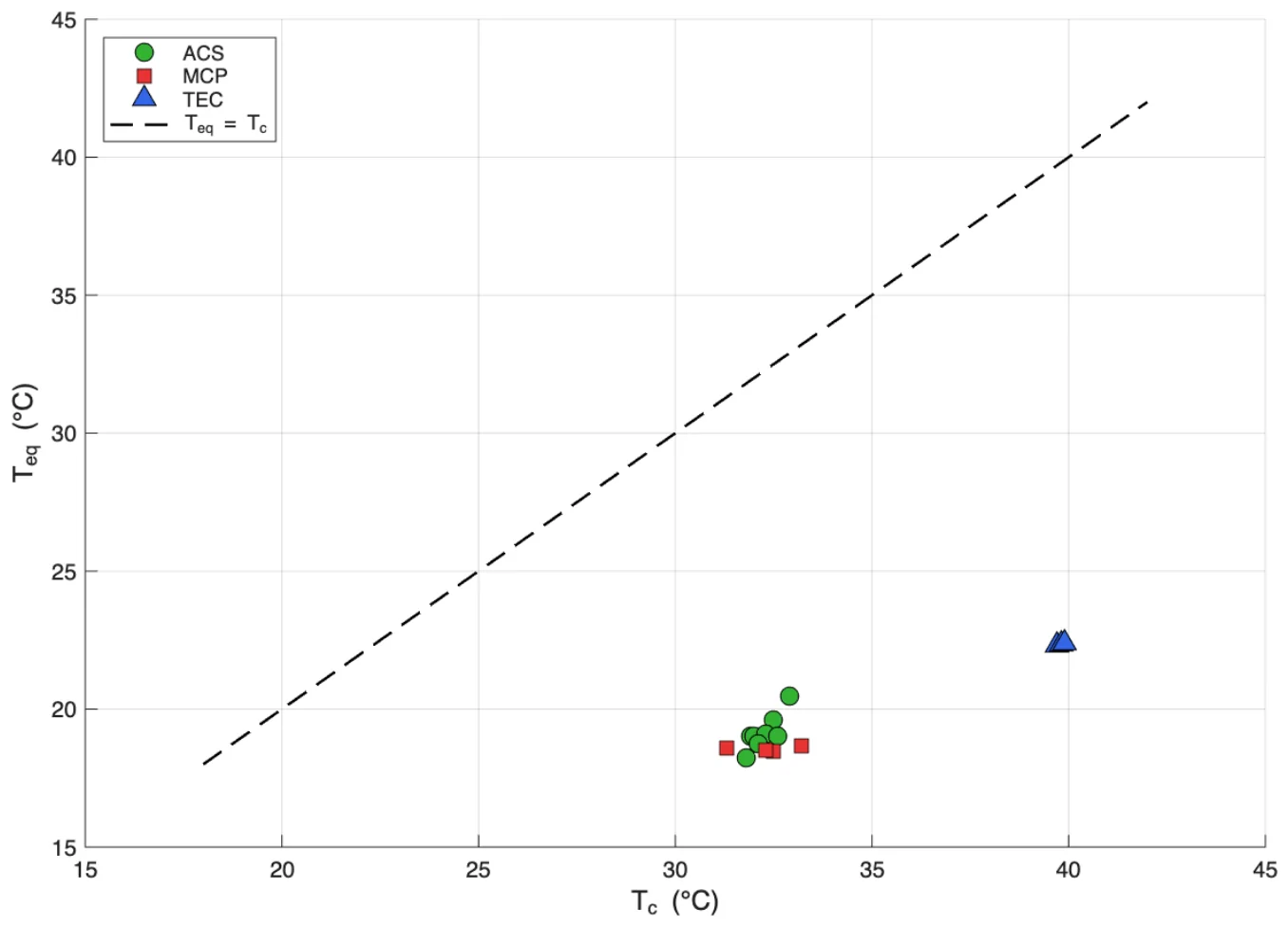
Thermomechanical correlation map between the equivalent temperature $T_{eq}$ and the viscoelastic transition temperature $T_{c}$ for all investigated IOL materials. ACS (green), MCP (red), and TEC (blue) lenses form distinct clusters reflecting their intrinsic relaxation dynamics. The diagonal line $T_{eq}=T_{c}$ marks the boundary between dissipative and transition regimes. ACS and MCP operate well below their transition temperature, whereas TEC lenses lie close to the diagonal, indicating that rapid folding drives these polymers toward a relaxation-limited state where elastic energy accumulation and edge damage become more likely.

The dioptric dependence of $T_{eq}$ discussed above shows how lens geometry modulates the strain-rate-induced thermomechanical shift. To synthesize these results within a broader materials-science perspective, Figure 5 compares the equivalent temperature $T_{eq}$ with the viscoelastic transition temperature $T_{c}$ for all investigated lenses. Although $T_{eq}$ is computed using $T_{c}$ as reference within the inverted WLF formulation, the correlation between the two quantities is not imposed mathematically. If the relationship were purely algebraic, all points would lie on the diagonal $T_{eq}=T_{c}$. Instead, the materials form distinct clusters, revealing that the strain-rate-induced thermomechanical shift is governed by the intrinsic relaxation dynamics of each polymer family.

ACS and MCP lenses occupy the lower-left region of the plot, where $T_{eq}$ remains well below $T_{c}$. Even under rapid folding, these polymers operate in a fully dissipative regime in which relaxation processes are sufficiently fast to accommodate the imposed deformation without significant elastic energy accumulation. Their large separation from the diagonal reflects a substantial thermomechanical safety margin: the strain-rate imposed by the injector is unable to drive the polymer close to its viscoelastic transition.

In contrast, TEC lenses cluster near the diagonal $T_{eq}=T_{c}$, indicating that injector-based folding drives these materials toward their viscoelastic transition. In this regime, the relaxation time becomes comparable to the characteristic deformation time, reducing the ability of the polymer network to dissipate strain energy. The proximity to the diagonal therefore represents a mechanically vulnerable condition in which localized bending and edge compression may exceed the relaxation capability of the material, favoring the onset of microcracks or chipping.

The overall trend across all materials shows a positive correlation between $T_{eq}$ and $T_{c}$, demonstrating that the strain-rate-induced thermomechanical shift is governed by the same molecular mechanisms that define the viscoelastic transition. Materials with higher $T_{c}$ exhibit higher $T_{eq}$, confirming that their intrinsic segmental mobility limits the extent to which rapid deformation can activate dissipative relaxation. The $T_{eq}$–$T_{c}$ map therefore provides a physically transparent criterion for assessing mechanical vulnerability: edge damage becomes likely when the equivalent temperature approaches the viscoelastic yield. In conclusion, although clinicians occasionally perform pre-warming of IOLs as an empirical clinical practice, the DMA measurement and model-based risk prediction proposed in the present work might represent a future point of reference and are developed from theoretical predictions of the temperature-dependent viscoelastic behaviour.

## 5. Conclusions

The combined DMA analysis and time–temperature mapping provide a unified framework for understanding how hydrophobic acrylic IOLs respond to the rapid deformation imposed by modern injectors. The viscoelastic transition temperature $T_{c}$ and the equivalent temperature $T_{eq}$ emerge as the two key descriptors of mechanical safety during folding. While $T_{c}$ reflects the intrinsic relaxation properties of each polymer network, $T_{eq}$ translates the injector strain rate into an effective thermomechanical state, revealing whether the material behaves in a rubbery, viscoelastic, or frozen regime.

By transforming the WLF coefficients to use $T_{c}$ as a reference and inverting the WLF relation, we quantified the thermal shift required for each IOL to match the imposed strain rate. The resulting $T_{eq}$ values show that the injector can effectively “cool down” or “warm up” the polymer depending on its geometry and material family. High $T_{eq}$ values (30–35 °C) indicate that the deformation is gentle enough for the polymer to remain fully relaxed, whereas $T_{eq}$ approaching $T_{c}$ signals that the strain rate consumes the thermal safety margin and drives the material toward its viscoelastic transition.

The thermomechanical correlation map presented in Figure 5 further clarifies this behavior by comparing $T_{eq}$ with $T_{c}$ across all materials [22]. Although $T_{eq}$ is computed using $T_{c}$ as reference, the correlation between the two quantities is not imposed by the WLF formulation. Instead, the three material families form distinct clusters, demonstrating that the strain-rate-induced thermomechanical shift is governed by the intrinsic relaxation dynamics of each polymer network. ACS and MCP lenses operate well below their transition temperature, whereas TEC lenses lie close to the diagonal $T_{eq}=T_{c}$, indicating that rapid folding drives these polymers toward a relaxation-limited regime where elastic energy accumulation becomes significant.

Integrating these thermomechanical descriptors with the geometric strain model yields the chipping index $I_{s}$, a dimensionless parameter that quantifies the ratio between the imposed deformation rate and the intrinsic relaxation capability of the polymer. The distribution of commercial IOLs across the three mechanical regimes defined by $I_{s}$ (safe, transition, chipping) reveals a clear material-dependent stratification: ACS and MCP lenses operate predominantly in the dissipative regime, whereas TEC lenses cluster near or above the transition threshold.

Although developed for hydrophobic acrylic IOLs, the thermomechanical framework established here is broadly applicable to polymer networks experiencing rapid deformation. The integration of DMA relaxation data, WLF-based equivalent temperature mapping, and strain-rate modeling offers a general strategy for identifying failure-sensitive regimes in soft materials. Future work may extend this approach to emerging biomaterials, alternative injector geometries, and polymer formulations with tailored relaxation spectra, enabling predictive design of safer and more robust polymeric devices.

## Figures and Tables

**Table 1:** Viscoelastic transition temperature $T_{c}$, crossover modulus $E^{\prime }=E^{\prime \prime }$, and equivalent temperature $T_{eq}$ for ACS, MCP, and TEC hydrophobic acrylic polymers.

Material	Diopter (m^−1^)	$T_{c}$ (°C)	$E^{\prime }=E^{\prime \prime }$ (MPa)	$T_{\mathrm{eq}}$ (°C)
ACS	15.0	32.90	0.613	20.49
ACS	18.5	32.50	0.375	19.59
ACS	19.5	31.90	0.760	19.02
ACS	20.0	32.00	0.400	19.01
ACS	21.0	32.30	0.544	19.08
ACS	22.5	32.10	0.645	18.74
ACS	23.0	32.60	0.333	19.02
ACS	25.0	31.80	0.731	18.24
MCP	18.0	32.50	0.651	18.47
MCP	18.5	31.30	0.924	18.60
MCP	21.5	32.30	0.480	18.51
MCP	23.5	33.20	0.162	18.66
TEC	20.0	39.70	0.826	22.32
TEC	21.0	39.80	0.745	22.36
TEC	22.0	39.90	0.672	22.40

## Data Availability

The original contributions presented in this study are included in the article. Further inquiries can be directed to the corresponding author.
